# Extremely Fast and Cheap Densification of Cu_2_S by Induction Melting Method

**DOI:** 10.3390/ma14237311

**Published:** 2021-11-29

**Authors:** Paweł Nieroda, Krzysztof Ziewiec, Juliusz Leszczyński, Paweł Rutkowski, Andrzej Koleżyński

**Affiliations:** 1 Department of Inorganic Chemistry, Faculty of Materials Science and Ceramics, AGH University of Science and Technology, al. A. Mickiewicza 30, 30-059 Krakow, Poland; jleszczy@agh.edu.pl; 2 Institute of Technology, Pedagogical University of Cracow, Podchorążych 2, 30-084 Krakow, Poland; kziewiec@up.krakow.pl; 3 Department of Ceramics and Refractories, Faculty of Materials Science and Ceramics, AGH University of Science and Technology, al. A. Mickiewicza 30, 30-059 Krakow, Poland; pawelr@agh.edu.pl; 4 Department of Silicate Chemistry and Macromolecular Compounds, Faculty of Materials Science and Ceramics, AGH University of Science and Technology, al. A. Mickiewicza 30, 30-059 Krakow, Poland; andrzej.kolezynski@agh.edu.pl

**Keywords:** copper(I) sulfide, thermoelectric materials, induction melting, thermoelectric properties

## Abstract

The aim of this work was to obtain dense Cu_2_S superionic thermoelectric materials, homogeneous in terms of phase and chemical composition, using a very fast and cheap induction-melting technique. The chemical composition was investigated via scanning electron microscopy (SEM) combined with an energy-dispersive spectroscopy (EDS) method, and the phase composition was established by X-ray diffraction (XRD). The thermoelectric figure of merit *ZT* was determined on the basis of thermoelectric transport properties, i.e., Seebeck coefficient, electrical and thermal conductivity in the temperature range of 300–923 K. The obtained values of the *ZT* parameter are comparable with those obtained using the induction hot pressing (IHP) technique and about 30–45% higher in the temperature range of 773–923 K in comparison with Cu_2_S samples densified with the spark plasma sintering (SPS) technique.

## 1. Introduction

Cu_2_S is an environmentally friendly and low-cost superionic thermoelectric material that has been intensively studied in recent years as a potential material for thermoelectric generator applications [1,2,3,4]. The energy conversion efficiency of such thermoelectric generators depends on the temperature difference between the junctions of the thermoelectric materials and the properties of materials expressed by the value of the thermoelectric figure of merit, *ZT* = *α*^2^*σλ*^−1^*T* [5,6], (*α*—Seebeck coefficient, *σ*—electrical conductivity, *λ*—thermal conductivity). The remarkable thermoelectric properties of Cu_2_S, both undoped and doped with e.g., Se [2], in particular for the high-temperature cubic phase α-Cu_2_S (*T* > 708 K) [7] are closely related to the migration of copper ions. Explanation of this phenomenon is provided by the phonon-liquid electron-crystal PLEC [8] concept, which is an extension of the phonon-glass electron-crystal PGEC concept [9] to ionic conductors. The migration of ions in the Cu_2_S crystal structure, on the one hand, has a positive effect on the thermoelectric properties, i.e., a decrease in thermal conductivity and an increase in the electrical conductivity of the material, but on the other hand causes a lack of stability of Cu_2_S under electric current. This may result in a deficiency of copper atoms in the Cu_2_S structure, which affects the transport properties of the material [10].

The most efficient and widely used sintering technique for thermoelectric materials is spark plasma sintering (SPS) [11] using a pulsed DC current. However, for materials with high ion mobility, which include superionic compounds, this technique does not seem to be suitable due to possible ion migration, resulting in a change in chemical composition of such materials [12]. In our previous work [13], we have shown that using the induction hot pressing (IHP) technique, it is possible to significantly improve the thermoelectric figure of merit *ZT* by up to 40% compared to Cu_2_S obtained via the SPS technique [14,15]. This improvement seems to be related to the way the samples are heated during sintering, i.e., without any current flowing through the sintered material [16]. However, both SPS and IHP techniques require the use of expensive equipment, which often limits their potentially wide range of applications. To address the latter problem, this work proposes a very fast and at the same time inexpensive method of Cu_2_S densification, which is based on the application of the induction melting (IM) technique.

**Figure 1:**
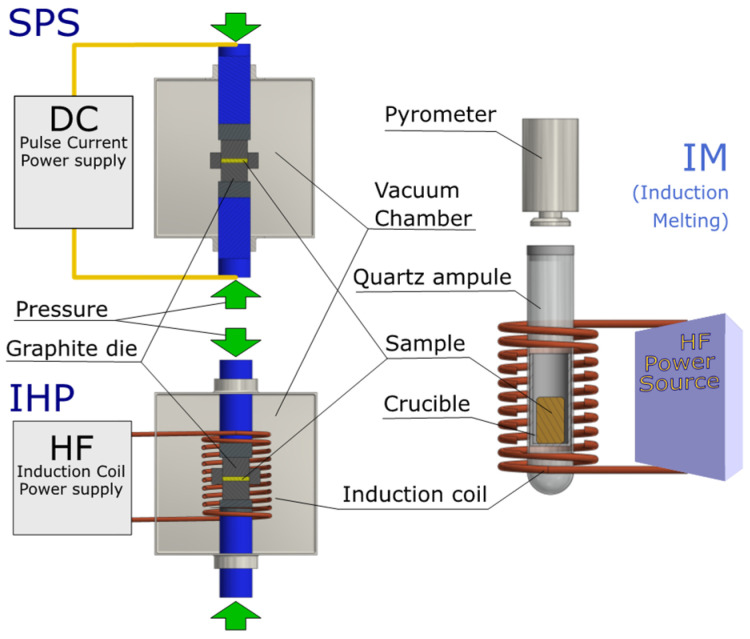
Schematic diagrams of SPS, IHP and IM techniques.

The principles of operation of SPS, IHP and IM methods is shown in Figure 1. The SPS and IHP methods use temperature and pressure to compact material. The powder material is placed in a graphite matrix, which, together with the powder, is heated by impulse current (SPS) or eddy current (IHP). Additionally, a pressure-generating force is applied to the punches of the die compressing the powder. The matrix is placed in a vacuum chamber, allowing anaerobic conditions, but this requires long flushing and pump-down times. These devices are quite large and expensive. Despite the fact that sintering takes quite a short time in both methods, much shorter than in the hot-pressing method, the time needed for preparation and cooling is several dozen minutes. The induction-melting method for densification uses both melting and crystallization. For this reason, induction melting is, in practice, applicable for congruent melting compounds. As in the IHP method, eddy currents are the source of heat, but they are generated in a small graphite crucible and directly in the melted powder. The advantage of these eddy currents is the mixing of the liquid melt, limiting the separation of the components. The powder is placed in a quartz ampoule with gas inlets, allowing it to be vacuum-filled or filled with an inert gas. Measurement and adjustment of the charge temperature are made contactless using a pyrometer, placed above the outlet of the ampoule, with a closed visor. This allows for better temperature control than in the case of SPS and IHP, where the temperature is measured with a thermocouple or with a pyrometer in a graphite die. The low heat capacity of such a system allows very fast heating and cooling rates of several thousand degrees per minute, so the process time can be very short. As a result, the growth of the grains can be limited and, in some cases, the high-temperature phase can be frozen if desired.

## 2. Materials and Methods

In this work, Cu_2_S samples, obtained from powders synthesized under three different conditions and then inductively melted, were obtained and studied. In the following part of the work, the designations IM1, IM2, and IM3 are used for the samples. In the first step, Cu (Alfa Aesar, Thermo Fisher (Kandel) GmbH, Kandel, Germany, 99.9%) and S (Alfa Aesar, Thermo Fisher (Kandel) GmbH, Kandel, Germany, 99.999%) powders were weighed in a glovebox in stoichiometric amounts and then encapsulated in a quartz ampule under vacuum. The synthesis was carried out via one- or two-step annealing. Sample IM1 was annealed at *T* = 673 K for *t* = 3 h and sample IM2 at *T* = 673 K for *t* = 24 h. In the first stage, sample IM3 was annealed at *T*_1_ = 673 K for *t*_1_ = 24 h and, after grinding the obtained ingot and resealing in a quartz ampoule, it was annealed at *T*_2_ = 1173 K for *t*_2_ = 18 h. The ingots obtained after synthesis were then ground in an agate mortar and remelted (*T* = 1423 K, *v* = 2500 K·min^−1^, *t* = 1 min) using the IM technique. The density of the obtained samples, measured by the hydrostatic Archimedes method was high and amounted to approx. 98% of the theoretical density (98.3 ± 0.2% for IM3, 97.9 ± 0.2% for IM2 and 97.4 ± 0.2% for IM1).

Both powders after synthesis and sinters were examined for phase composition by X-ray diffraction (X-ray Diffractometer Panalytical Empyrean, Malvern, Worcestershire, United Kingdom), CuKα, λ = 1.5418 Å, 2Θ angle range from 10° to 137°) at room temperature and, due to the high number of reflections at room temperature and the resulting difficulty in identification of all phases present in the system, additionally at 493 K. The chemical composition and microstructure of the specimens after melting were determined using a scanning electron microscopy SEM technique (FEI Nova NanoSEM 200 FEI Europe Company Scanning Electron Microscope, FEI COMPANY, Hillsboro, OR, United States) equipped with an EDAX detector.. Electrical conductivity and the Seebeck coefficient were studied in the temperature range of 300–923 K, using a homemade apparatus. Electrical conductivity was studied using a four-probe method with variable DC polarization. Seebeck coefficient measurements were carried out using a small (ΔT < 3 K), forced-temperature gradient across the specimen. Thermal diffusivity and heat capacity (Cp) were measured using a laser flash method LFA (Laser Flash Apparatus (LFA) 427, Netzsch, Selb, Germany) in the temperature range of 300–920 K (Figures S1 and S2 in the Supplementary Materials, respectively). Thermal conductivity was calculated from the relationship: λ = α·Cp·ϱ, where ϱ represents the density.

## 3. Results

### 3.1. Structural and Microstructural Analysis

**Figure 2:**
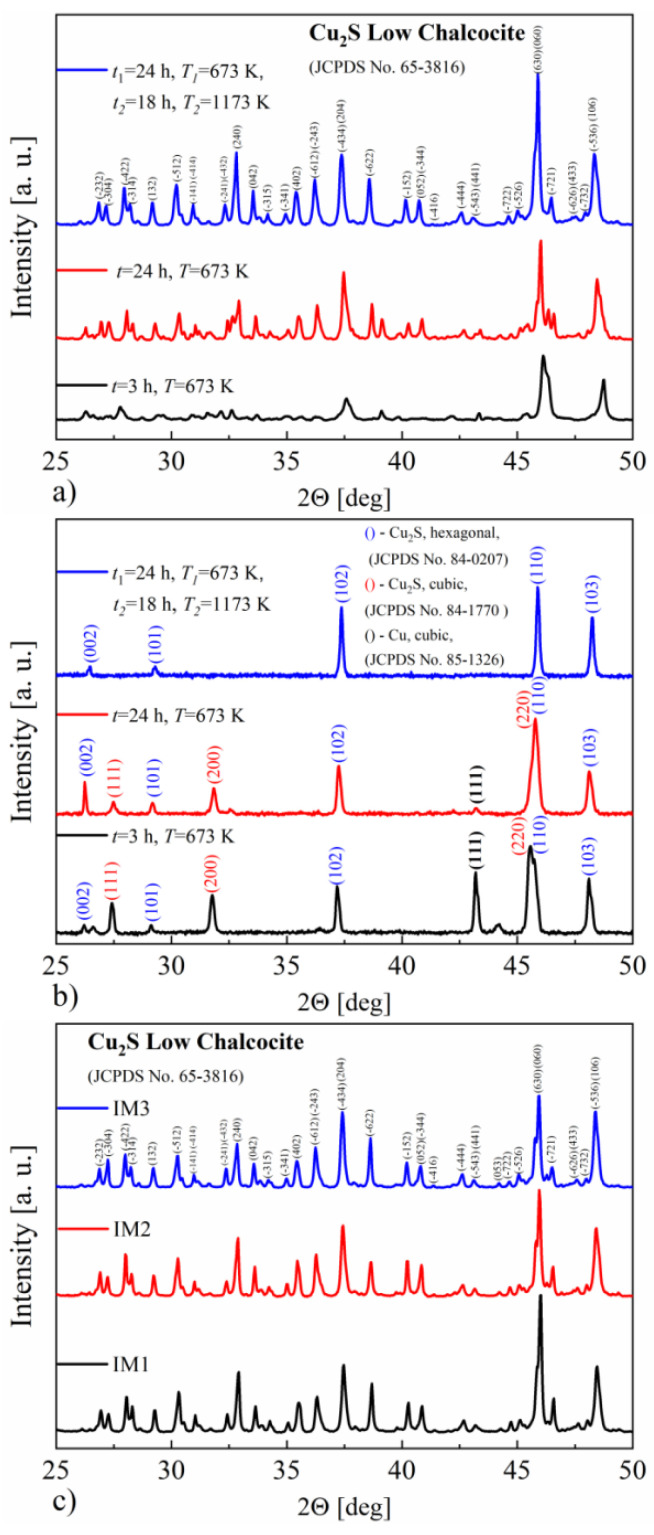
X-ray diffraction patterns for Cu_2_S samples synthesized under various conditions, measured at (**a**) RT and (**b**) 493 K, and (**c**) additionally measured at RT for the samples after induction-melting densification.

Figure 2a shows the XRD patterns obtained at room temperature for the Cu_2_S specimens. All obtained samples consist mainly of the monoclinic Cu_2_S phase. As can be seen, extending the synthesis time and increasing the temperature allows obtaining a better-crystallized form of the material. The precipitations of other phases, including copper, are not visible on the recorded diffraction patterns, which may be due to a large number of reflections in the monoclinic Cu_2_S phase. Larger differences between the samples are revealed in the XRD measurements at the temperature of 493 K, i.e., above the phase transition temperature of the Cu_2_S monoclinic to hexagonal phase [7,17] (Figure 2b). There is a clear difference in phase composition between the samples obtained at 673 K and the sample also annealed at 1173 K, which comprises the pure hexagonal copper sulfide phase. The samples obtained by annealing at 673 K contain two Cu_2_S phases—cubic and hexagonal. Additionally, the presence of copper can be observed, the amount of which clearly decreases with increasing synthesis time. After induction melting of the synthesized Cu_2_S, the XRD studies presented in Figure 2c show that, at room temperature, the resulting materials still consist mainly of a monoclinic phase. For samples IM1 and IM2, the observed XRD patterns reveal a much better crystallinity of the obtained material relative to the post-synthesis state, equal to that of sample IM3.

**Figure 3:**
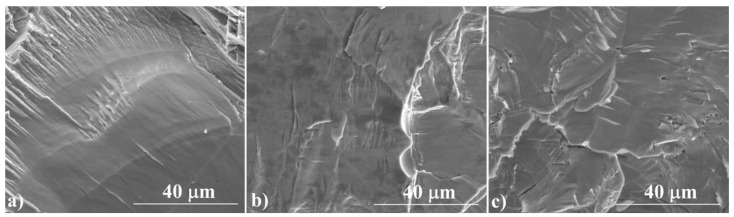
SEM photographs of the surface of cross-section fractures in the Cu_2_S samples (**a**) IM1, (**b**) IM2, (**c**) IM3, at a magnification of 3000×.

**Figure 4:**
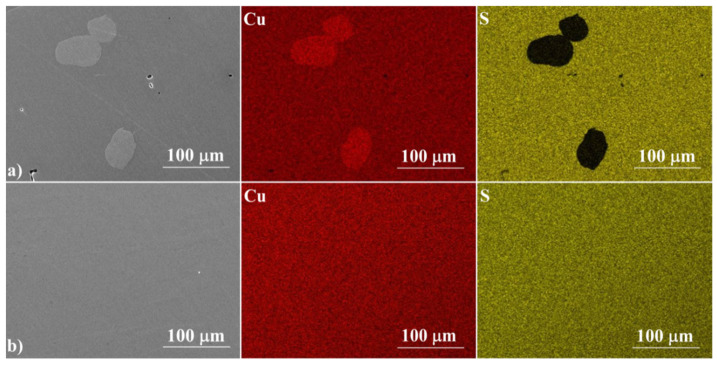
SEM photographs of a selected surface, with respective maps of element distribution for (**a**) IM2 and for (**b**) IM3.

Figure 3 shows the cross-sections of the samples, which confirm their high degree of densification, with no visible pores or voids. For a sample annealed for 3 h at 673 K, the fracture resembles a glassy material, while a sample annealed at 1173 K shows the features of a polycrystalline material. The EDS analysis (Figure 4) confirms the results of the XRD measurements. The ratio of Cu to S is close to 2:1 and is fairly homogeneous over the whole analyzed area. Similarly to XRD measurements, Cu precipitates are observed in samples synthesized exclusively at 673 K. These observations show that the induction melting process is so fast that the kinetics of the processes occurring in the melt are too slow to homogenize the melted and crystallized material. This indicates the necessity of using well-reacted and homogenized Cu_2_S as a starting material in this type of densification method, which, as we have shown, can be achieved by allowing a sufficiently long 24-hour synthesis stage at 1173 K.

### 3.2. Thermoelectric Transport Properties

**Figure 5:**
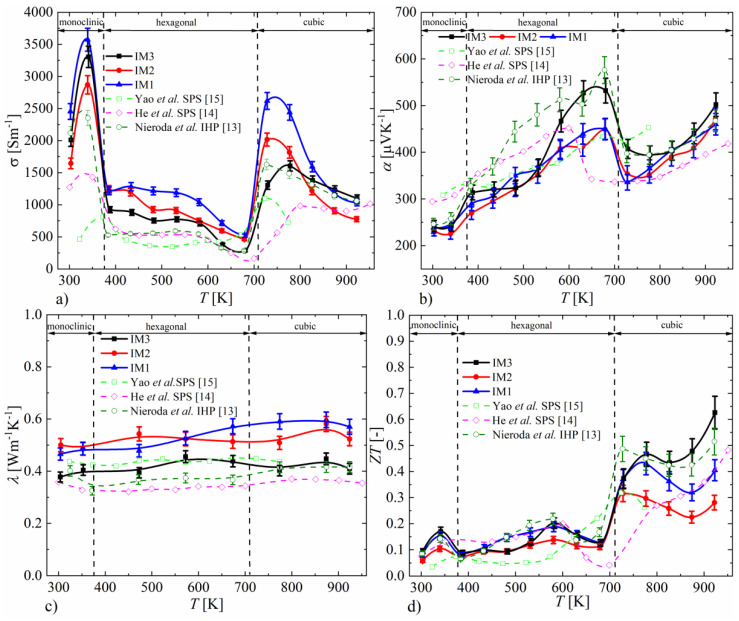
Temperature dependence of electrical conductivity (**a**), Seebeck coefficient (**b**), thermal conductivity (**c**), and *ZT* parameter (**d**) for Cu_2_S samples (absolute errors equal to 5% for α, σ, λ and 10% for *ZT*).

The electrical conductivity measurements are shown in Figure 5a. For all the samples, temperature dependence follows a fairly typical form, with a clearly visible decrease in conductivity for the hexagonal phase. In general, the conductivity of the melted samples is higher than that reported in the literature, which is particularly visible in the temperature range specific to the cubic phase. It can be seen that the longer the synthesis time and the smaller the number of foreign phases, the lower the Cu_2_S conductivity. In view of the results obtained, this increase in conductivity can be explained by the presence of Cu precipitates, which should be accompanied by the nonstoichiometry of the material. For the sample after induction-remelting that has the highest purity and homogeneity, the conductivity values are very close to our previous results for the material compacted by the IHP method. The results of the Seebeck coefficient measurements (Figure 5b) also agree well with the literature data. In the entire temperature range, the Seebeck coefficient takes positive values, and a step-change in its value can be observed near the phase transition temperatures [18]. For the sample annealed at 1173 K, the Seebeck coefficient values are slightly higher, especially near the temperature of transformation of the hexagonal to cubic phases. As in the case of electrical conductivity, the results obtained for this sample agree very well with the results of our previous studies [13]. The thermal conductivity (Figure 5c) varies little with temperature and does not deviate from the literature data. The total thermal conductivity is slightly higher for samples synthesized exclusively at 673 K, which is related to their higher electrical conductivity.

The thermoelectric figure of merit, *ZT* (Figure 5d), similarly to electrical conductivity and the Seebeck coefficient, noticeably changes during phase transitions between individual Cu_2_S structures. For the monoclinic and hexagonal phases, the *ZT* values vary between 0.05 and 0.2. A several-times-higher *ZT* of Cu_2_S is observed after transformation to the cubic phase. The temperature dependence of *ZT* for the cubic phase is non-monotonic. As the temperature increases, the *ZT* decreases, reaching a minimum at a temperature dependent on the method of synthesis, after which it increases. In the case of samples IM1 and IM2, this minimum is at a temperature around 870 K, and the maximum *ZT* value is reached at a temperature below this minimum except in the cubic phase. For sample IM3, the *ZT* minimum for the cubic phase is at around 820 K; the maximum *ZT* value is observed at 920 K and reaches around 0.6, which is the highest value for all samples obtained in this work. The improved thermoelectric properties of sample IM3 with respect to samples IM1 and IM2 are mainly due to the lower thermal conductivity and the smaller decrease in electrical conductivity with increasing temperature. These more favorable properties are related to the greater homogeneity of the material synthesized at 1173 K, expressed by the absence of copper precipitates and the more favorable, more stoichiometric composition of Cu_2_S. The temperature dependence of *ZT*, determined for the materials obtained by induction melting, is similar to the results presented in the literature [13,14,15]. The most comparable properties can be observed for the sample obtained by IHP sintering in our previous studies [13], which has a very similar temperature dependence and values of *ZT* as for the IM3 sample. However, the thermoelectric properties are better than those of SPS-densified samples synthesized both by direct high-temperature synthesis and by mechanical alloying, which may be due to their degradation during the SPS process resulting from the current flow through the material.

## 4. Conclusions

In conclusion, we have shown in the current work that by using induction remelting, it is possible to obtain dense and homogeneous Cu_2_S. The IM (induction melting) method offers very short compaction times, and the remelting time is so brief that the mixing and segregation processes have virtually no time to take place. Therefore, it is preferable to use materials of high purity and phase homogeneity as starting materials for densification. The obtained samples have a higher value of electrical and thermal conductivity than materials densified using the SPS and IHP methods. This results in significantly higher *ZT* values than for materials compacted by the SPS method, and slightly higher than those for materials compacted by the IHP method. The inferior properties of SPS-sintered materials may be due to the degradation of the material subjected to direct current flow, which does not occur in the case of IHP sintering and IM densification. Since the IM method proposed in this work is much faster than the SPS and IHP methods and, simultaneously, does not require such expensive equipment, it can find a wide application in obtaining other bulk materials, not only thermoelectrics, that melt congruently.

## Data Availability

Data sharing is not applicable to this article.

## Supplementary Materials

The following are available online at https://www.mdpi.com/article/10.3390/ma14237311/s1, Figure S1: Temperature dependence of thermal diffusivity for Cu_2_S samples, Figure S2: Temperature dependence of specific heat for Cu_2_S samples.
