# Supplementary material for: Extremely Fast and Cheap Densification of Cu2S by Induction Melting Method
Source: Materials (Basel). 2021 Nov 29;14(23):7311. doi: 10.3390/ma14237311 (PMC8658081; doi:10.3390/ma14237311)
Supplement: Supplementary file 1 [file materials-14-07311-s001.zip › materials-1469807-supplementary.pdf]

Supplementary material

Extremely Fast and Cheap Densification of Cu_2S by Induction Melting Method

Paweł Nieroda[1,*], Krzysztof Ziewiec[2], Juliusz Leszczyński[1], Paweł Rutkowski[3] and Andrzej Koleżyński[4]

[1] Department of Inorganic Chemistry, Faculty of Materials Science and Ceramics, AGH University of Science and Technology, al. A. Mickiewicza 30, 30-059 Krakow, Poland; jleszczy@agh.edu.pl (J.L.)

[2] Institute of Technology, Pedagogical University of Cracow, Podchorążych 2, 30-084, Krakow, Poland; kziewiec@up.krakow.pl (K.Z.)

[3] Department of Ceramics and Refractories, Faculty of Materials Science and Ceramics, AGH University of Science and Technology, al. A. Mickiewicza 30, 30-059 Krakow, Poland; pawelr@agh.edu.pl (P.R.)

[4] Department of Silicate Chemistry and Macromolecular Compounds, Faculty of Materials Science and Ceramics, AGH University of Science and Technology, al. A. Mickiewicza 30, 30-059 Krakow, Poland; andrzej.kolezynski@agh.edu.pl (A.K.)

* Correspondence: pnieroda@agh.edu.pl

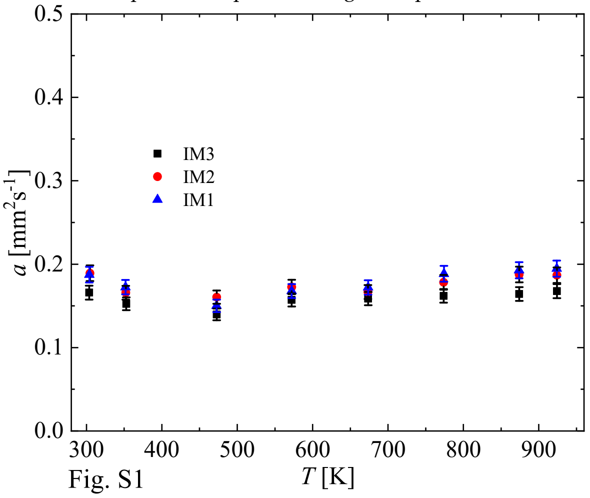

Figure S1. Temperature dependence of thermal diffusivity for Cu_2S samples.

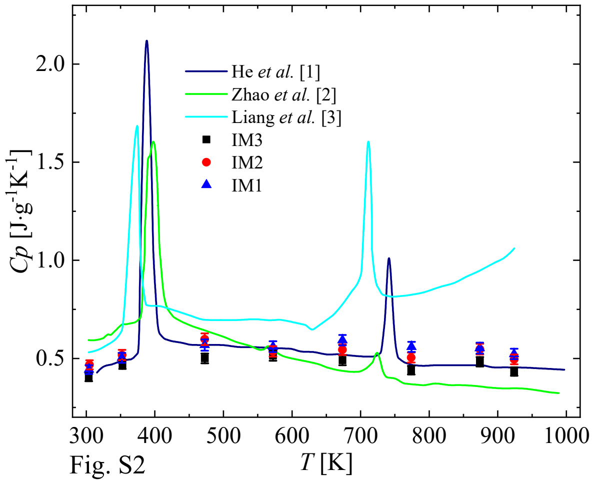

Figure S2. Temperature dependence of specific heat for Cu_2S samples.

[1] Y. He, T. Day, T. Zhang, H. Liu, X. Shi, L. Chen, and J.G. Snyder, Adv. Mater. 26, 3974, (2014).

[2] L. Zhao, X. Wang, F.Y. Fei, J. Wang, Z. Cheng, S. Dou, J. Wang and G.J. Snyder, J. Mater. Chem. A, 3, 9432, (2015).

[3] X. Liang, Appl. Phys. Lett. 111, 133902, (2017).
